# A Two-Year Surveillance of 2009 Pandemic Influenza A (H1N1) in Guangzhou, China: From Pandemic to Seasonal Influenza?

**DOI:** 10.1371/journal.pone.0028027

**Published:** 2011-11-18

**Authors:** Tiegang Li, Chuanxi Fu, Biao Di, Jibin Wu, Zhicong Yang, Yulin Wang, Meixia Li, Jianyun Lu, Yiyun Chen, Enjie Lu, Jinmei Geng, Wensui Hu, Zhiqiang Dong, Meng-feng Li, Bo-Jian Zheng, Kai-yuan Cao, Ming Wang

**Affiliations:** 1 Guangzhou Center for Disease Control and Prevention, Guangzhou, China; 2 Department of Microbiology, Zhongshan School of Medicine, Sun Yat-sen University, Guangzhou, China; 3 Key Laboratory of Tropical Disease Control, Ministry of Education, Sun Yat-Sen University, Guangzhou, China; 4 University of Hong Kong, Hong Kong SAR, China; University of Cambridge, United Kingdom

## Abstract

In this two-years surveillance of 2009 pandemic influenza A (H1N1) (pH1N1) in Guangzhou, China, we reported here that the scale and duration of pH1N1 outbreaks, severe disease and fatality rates of pH1N1 patients were significantly lower or shorter in the second epidemic year (May 2010-April 2011) than those in the first epidemic year (May 2009-April 2010) (*P*<0.05), but similar to those of seasonal influenza (*P*>0.05). Similar to seasonal influenza, pre-existing chronic pulmonary diseases was a risk factor associated with fatal cases of pH1N1 influenza. Different from seasonal influenza, which occurred in spring/summer seasons annually, pH1N1 influenza mainly occurred in autumn/winter seasons in the first epidemic year, but prolonged to winter/spring season in the second epidemic year. The information suggests a tendency that the epidemics of pH1N1 influenza may probably further shift to spring/summer seasons and become a predominant subtype of seasonal influenza in coming years in Guangzhou, China.

## Introduction

During the spring of 2009, the Centers for Disease Control and Prevention (CDC) confirmed the first two cases of human infection with the 2009 pandemic influenza A (H1N1) (pH1N1) virus in the USA [1], [2]. As the virus spread rapidly to other regions of the world [2], [3], [4], the World Health Organization (WHO) declared the first phase VI global influenza pandemic on 11 June 2009. By 25 July 2010, worldwide more than 214 countries and overseas territories or communities had reported laboratory confirmed pH1N1 cases, including 18398 deaths [5].

Guangzhou is the largest trading city in southern China with over 7.94 million registered inhabitants and 4.76 million floating population. Considering that pH1N1 influenza was inevitable to spread to Guangzhou, we conducted a surveillance system of pH1N1 influenza in early May 2009 in Guangzhou, before the first imported pH1N1 influenza case (from the US) in China was reported on 18 May 2009. Subsequently, we reported the first confirmed native pH1N1 case [6], the first outbreak of pH1N1 and the first severe pH1N1 case [7] in China in May, July and August 2009, respectively.

Although WHO declared that the new H1N1 virus has largely run its course and the world has been moving into the post-pandemic period on 10 August 2010 [8], we continued our surveillance of pH1N1 influenza in Guangzhou and analyzed the data based on information provided by four general surveillance hospitals and twelve regional CDC during the two-years surveillance period from May 2009 to April 2011. Large numbers of studies/investigations on epidemiology and clinical features of the first epidemic year of pH1N1 influenza from 2009 to 2010 have been reported [3], [9], [10], [11], [12]. However, only little information is available for the second epidemic year of pH1N1 influenza from 2010 to 2011 [13]. In this paper, we described the information obtained from a two years surveillance of pH1N1 influenza in Guangzhou from May 2009 to April 2011, and compared with those of traditional seasonal influenza in order to detect the epidemic tendency of pH1N1 and assist public health control measures.

## Materials and Methods

The protocol of this surveillance was approved by the ethics committee of Guangzhou Center for Disease Control and Prevention (GZCDC) and informed consent was obtained from all subjects (or children's guardians) recruited to receive a swab or blood test.

### Outbreaks of pH1N1 influenza

When the number of fever cases with influenza-like symptoms increased to over 3 per day or 5 per 3 consecutive days in the same dormitory, classroom or office in Guangzhou, the people in charge of team health care collected basic information and reported to the regional CDC by telephone or fax. Technical staff in regional CDC conducted field investigation and verification. Throat swab samples from at least 10 of these cases were collected and sent to GZCDC for pH1N1 virus and other influenza virus confirmation [9]. An outbreak was identified as that at least 15 cases/week were found in any school, kindergarten or other group. The attack rate was defined as (Total number of new cases in the observation period/Number of exposed population at the same period)×100. The scale was defined as average attack rate/outbreak (%).

### Virological surveillance

Four general hospitals and twelve regional CDC, located in 12 districts of Guangzhou City, were selected as the surveillance spots. Four general hospitals were selected because they are the four largest hospitals in the Guangzhou, which located in the center of the city and and they are the first choice hospital for the public after suffering from illness. In the day out-patient clinics of these four hospitals, the patients who appeared body temperature ≥ 38°C accompanying with cough or sore throat symptoms were identified as suspected cases of influenza. Serum and/or swab samples were collected from these suspected cases for further virological surveillance.

The presence of influenza virus in swab samples was detected by isolation of the virus in cell cultures and/or real-time RT-PCR, while antibodies specific to influenza virus were tested by HAI and/or ELISA, as described previously [8], [9], [14]. Influenza cases were confirmed by at least one of positive results of following laboratory tests: i) isolation of the virus; ii) RNA of influenza virus, including seasonal H1N1, H3N2, B, and pH1N1; and iii) seroconversion of influenza specific antibodies.

### Severe cases

According to the criteria of Diagnosis and Treatment Guideline for Influenza issued by China Ministry of Health in 2009, severe cases of influenza were identified as patients who appeared: i) persistent fever lasting for > 3 days accompanying with severe cough, ii) purulent sputum, bloody sputum, or chest pain, iii) respiration frequency becoming faster, difficult breathing, cyanosis of lips, iv) mind changes, such as slow, drowsiness, restlessness, convulsions, etc, v) severe vomiting, diarrhea, dehydration performance, vi) imaging signs of pneumonia, vii) creatine kinase (CK), creatine kinase-MB (CK-MB) and/or other enzyme levels increased rapidly, viii) the conditions of original underlying diseases were significantly worse.

### Data analysis

Data were analyzed using SPSS statistical software (version 11.5, SPSS, Inc., Chicago, IL). χ^2^ test and/or Fisher exact test were/was used to compare proportions of different groups. T test was used to compare the difference between the groups. Binary logistic regression was employed to explore risk factors potentially associated with fatal pH1N1 cases, of which different pre-existing chronic diseases were considered as the independent variables. The progressions of severe pH1N1 cases were considered as the dependent variation, with 0 for recovered developing into mild pH1N1 patients and 1 for dead patients. For all analysis, *P* values ≤ 0.05 were regarded as significant.

## Results

### Outbreaks of pH1N1 influenza in Guangzhou

As shown in Table 1, a total of 208 pH1N1 outbreaks were reported from May 2009 to April 2011. Most outbreaks (205/208, 98.6%) occurred in the first epidemic year from May 2009 to April 2010, whereas only 3 outbreaks (1.4%) were found in the second epidemic year from May 2010 to April 2011. The frequency of outbreaks (average number of pH1N1 outbreaks/month) in the second epidemic year was 0.25, which was significantly lower than that (17.08) in the first epidemic year (*P*<0.05). The scale (average attack rate/outbreak) and duration (average days/outbreak) of pH1N1 influenza in the second epidemic year were 2.13% and 18.57 days, respectively, which were significantly lower or shorter than those (9.82% and 26.48 days) in the first epidemic year (*P*<0.05). However, the frequency, scale and duration of pH1N1 influenza outbreaks in the second epidemic year were similar to those of seasonal influenza outbreaks (*P*>0.05), suggesting that pH1N1 influenza had most likely become a seasonal influenza in the second epidemic year in Guangzhou.

**Table 1:** The frequency, scale and duration of outbreaks of influenza in the first and second epidemic year.

	1^st^ epidemic year	2^nd^epidemic year		(a) vs (b) *P*	(c) vs (b) *P*
	pH1N1 (a)	pH1N1 (b)	Seasonal (c)		
Frequency*	17.08 (205/12)	0.25 (3/12)	0.25 (3/12)	<0.05	1.00
Scale&	9.82 (31025/316029)	2.13 (19/890)	2.06 (602/29171)	<0.05	0.88
Duration#	26.48±7.11	18.57±5.77	14.00±2.00	<0.05	0.19

*Average number of outbreaks/month.

& Average attck rate/outbreak (%).

# Average days/outbreak±SD.

### Severe and fatal cases of pH1N1 influenza

**Figure 1:**
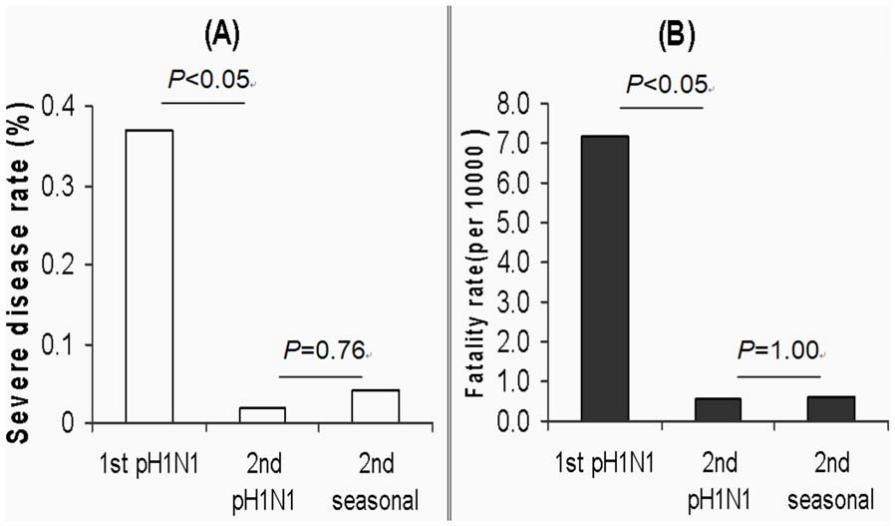
Severe disease and fatality rates of pH1N1 and seasonal influenza in Guangzhou. (A) Severe disease rate (% of patients progressed to severe disease) of pH1N1 influenza in the first (1st pH1N1) and second (2nd pH1N1) epidemic year and of seasonal influenza in the second epidemic year (2nd seasonal). (B) Fatality rate (deaths/10000) of pH1N1 influenza in the first (1st pH1N1) and second (2nd pH1N1) epidemic year and of seasonal influenza in the second year (2nd seasonal).

A total of 81 severe pH1N1 cases were reported during the surveillance period, of which 16 died. As shown in Fig 1, in the first epidemic year, the severe disease rate (% of patients progressed to severe disease) and fatality rate (deaths/10000) in pH1N1 influenza patients were 0.37% (77/20885) and 7.18 per 10000 (15/20885), respectively, whereas both decreased to 0.02% (4/17188) and 0.58 per 10000 (1/17188) in the second epidemic year. Both were significantly lower than those of pH1N1 influenza in the first epidemic year (*P*<0.05), but similar to those of seasonal influenza during the same period (*P*>0.05). Fatal cases and severe appeared in each age group ranging from 1 to 71 years old. In age groups of 0∼5, 6∼15, 16∼25, 26∼40, 41∼59 and 60∼ years, the fatal cases /severe cases/total cases were 6/27/2565, 1/6/18084, 3/14/11716, 2/13/3793, 4/17/1686, 0/4/229, respectively. The death rate of severe cases did not show significant difference between these age groups (*P*>0.05). Also, no pregnant patients were found in severe and fatal cases (data not shown). More than half of severe pH1N1 cases (43/81) had at least one pre-existing chronic disease. Potential risk factors of pre-existing chronic diseases associated with death were further analyzed using univariate analysis based on 77 severe cases reported in the first epidemic years (Table 2). The results showed that chronic pulmonary diseases were the risk factor associated with the death of severe pH1N1 influenza (OR = 6.00, 95%CI = 1.70–21.22), whereas no other chronic diseases were found to be associated with the death. In the 2^nd^ epidemic year, only 4 pH1N1 severe cases were reported and one of them dead and the fatal case also had pre-existing chronic pulmonary disease.

**Table 2.Univariate:** analysis of potential risk factors associated with the death of pH1N1 influenza severe cases in the first epidemic year.*

Factors	Into mild			Into death		OR#	95%CI
	+	−		+	−		
Chronic lung disease	7	54		7	9	6.00	1.70–21.22
Cardiovascular disease	7	54		3	13	1.78	0.40–7.83
Malignant tumor	1	60		2	14	8.57	0.73–101.04
Chronic renal disease	0	61		1	15	1.07	0.94–1.21
Nervous system disease	1	60		1	15	4.00	0.20–67.71
Immune system disease	1	60		1	15	4.00	0.20–67.71
Metabolism system disease	2	59		0	16	0.97	0.92–1.01
Hematological system Disease	1	60		1	15	4.00	0.20–67.71

*Four pH1N1 sever cases were reported in the second epidemic year with one death and the dead case also had pre-existing chronic pulmonary disease.

# OR = odds ratio.

### Seasonal distribution of pH1N1 influenza in Guangzhou

**Figure 2:**
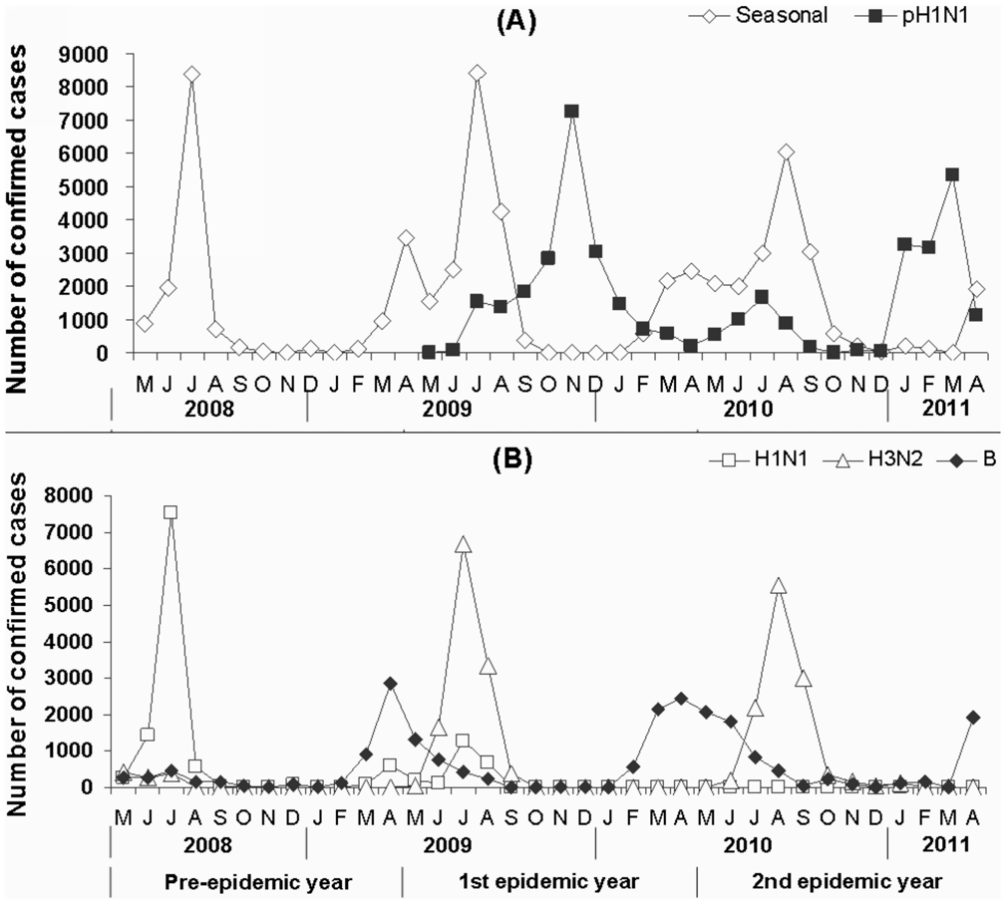
Seasonal distribution of influenza in Guangzhou from May 2008 to April 2011. (A) Confirmed cases of seasonal influenza (Seasonal, including H1N1, H3N2 and B) and pH1N1 influenza are shown in the indicated time. (B) Confirmed cases of seasonal influenza caused by subtypes H1N1, H3N2 and B are shown in the indicated time.

As shown in Figure 2A, in the first epidemic year, the epidemic of pH1N1 influenza started at June 2009 and lasted to April 2010, but mainly occurred in autumn/winter season from October to December and peaked in November 2009. In the second epidemic year, the major epidemic of pH1N1 influenza occurred in winter/spring seasons from January to April 2011 and peaked in March. Compared to seasonal influenza, which occurred in spring (B subtype) and summer (H1N1 and H3N2 subtypes) seasons (Figure 1B), epidemics of pH1N1 influenza seems to occur preferably in cold seasons. It should be noted, however, that the peak of pH1N1 epidemic had prolonged from November 2009 in the first epidemic year to March 2011 in the second epidemic year, suggesting a tendency that season of pH1N1 influenza epidemic may probably further shift to spring/summer seasons. Interestingly, seasonal influenza H1N1 virus was a predominant subtype resulting in the epidemic of influenza in the pre-epidemic year from May to August 2008, but became a subordinate subtype of epidemic of seasonal influenza in summer of 2009 and disappeared completely thereafter. Instead, pN1N1 virus, as a subordinate subtype, was co-circulating with seasonal influenza H3N2 virus in summer season. The information suggested that pH1N1 will probably replace the traditional seasonal influenza H1N1 and result in seasonal influenza in coming years in Guangzhou.

## Discussion

At the early stage of pH1N1 influenza spread to Guangzhou (May to June 2009), only a few persons acquired the infection (Fig. 2A), which might be attributed to the strict measures applied in Guangzhou for the control of this infectious disease. However, similar to the other countries and regions [15], [16], pH1N1 influenza virus was spread to the whole city and resulted in large number of outbreaks in the first epidemic year after August 2009 (Table 1), which is markedly higher than previous outbreaks of seasonal influenza in Guangzhou [17]. Consistently, the frequency, scale and duration of the outbreaks of pH1N1 influenza were significantly higher or longer than that of seasonal influenza, as we reported previously, in a boarding school the infection rate was 32% [18]. This might be due to the fact that pH1N1 virus was newly derived originally from swine and there was no or little immunity against the infection caused by this virus in the community of Guangzhou. However, we found that persons aged ≥ 60 years was only accounted for 0.60% of the total pH1N1 confirmed cases, which was similar to many other countries, such as the United States and England where less cases among people over 60 was also observed [19], [20], which needs further study.

Similar to other reports [15], [16], [21], [22], [23], [24], in Guangzhou, severe disease and fatality rates of pH1N1 influenza in the first epidemic year were significantly higher than those of seasonal influenza (Fig. 1), but significantly lower than the initial epidemic estimate [25]. However, it is different from previous reports that age, pregnancy, diabetes and obesity might be risk factors associated with severe and fatal cases of pHN1 influenza [26], [27], [28], [29], [30], [31], our results did not show significant association between these factors and severe/fatal cases of pH1N1 in Guangzhou (Table 2). Nevertheless, our results showed that, over half of severe cases had pre-existing chronic diseases, of which chronic pulmonary and cardiovascular diseases were more common, and only chronic pulmonary disease was the risk factor associated with the death of severe pH1N1 influenza. This is consistent with previous studies on seasonal influenza and some recent studies on pH1N1 influenza [26], [32], [33]. Our surveillance showed that features of pH1N1 influenza in the second epidemic year had been changed as follows: (1) Outbreaks of pH1N1 influenza reduced significantly from 205 in the first epidemic year to 3 in the second epidemic year. (2) The frequency, scale and duration of pH1N1 outbreaks in the second epidemic year were significantly lower or shorter than those in the first epidemic year. (3) Severe disease and fatality rates of pH1N1 influenza in the second epidemic year were significantly lower than those in the first epidemic year. These features of pH1N1 influenza in the second epidemic year are similar to those of seasonal influenza but significantly different from those of pH1N1 influenza in the first year. These changes of pH1N1 influenza in the second epidemic year may probably be attributed to part of the population in Guangzhou had experienced pH1N1 influenza in the first epidemic year or received pH1N1 influenza vaccination which was started in Guangzhou from October 2009 [34]. Although it has been reported that vaccination of 2009 pH1N1 influenza was effective [35], it has been reported that some persons were still infected by pH1N1 virus after they received the vaccination [34]. This may be due to: (1) as we have reported, over half of recovered patients and vaccinated persons would have lost sufficient immunity against the recurrence of the viral infection after half a year (17); and (2) influenza virus genome can mutate rapidly to escape the host immunity. Recently, similar changes in pH1N1 influenza epidemics are also reported by other groups [36]. Therefore, we may conclude that pH1N1 no longer caused a pandemic after the first epidemic year and had likely become a seasonal influenza in Guangzhou, China.

Our results also showed that, in Guangzhou, seasonal influenza occurred in spring (B subtype) and summer (H1N1 and/or H3N2 subtypes) seasons annually, whereas pH1N1 influenza mainly occurred in autumn/winter seasons from October to December 2009 in the first epidemic year (Fig. 2). The different seasonal distribution of seasonal influenza and pH1N1 influenza has also been reported by other studies [37], [38]. Notably we found that the epidemic of pH1N1 influenza had prolonged to winter/spring season from January to March 2011 in the second epidemic year, showing a tendency that the epidemics of pH1N1 influenza may probably further shift to spring/summer seasons (Fig. 2A). It is also noted that seasonal influenza H1N1 virus caused the epidemic of influenza in 2008, whereas H3N2 influenza became to be predominant in 2009 and 2010 accompanied with disappearance of seasonal influenza H1N1 after winter season of 2009.

Taken together, the information has suggested that pH1N1 influenza virus may probably replace the traditional seasonal influenza H1N1 resulting in seasonal influenza in coming years in Guangzhou. However, it is needed to be further confirmed in future years. Thus, we will continue our surveillance of pH1N1, practically in virological surveillance to monitor potential possible virus variations due to the viral gene mutations and reassortments.

## Acknowledgments

All enrollees participating in the surveillance are appreciated. Our special thanks to the public unit coordinators and nurses in influenza surveillance hospitals in Guangzhou.
